# Supplementary material for: Affinity Tag Coating Enables Reliable Detection of Antigen-Specific B Cells in Immunospot Assays
Source: Cells. 2021 Jul 21;10(8):1843. doi: 10.3390/cells10081843 (PMC8394687; doi:10.3390/cells10081843)
Supplement: Supplementary file 1 [file cells-10-01843-s001.zip › cells-1265064-supplementary.pdf]

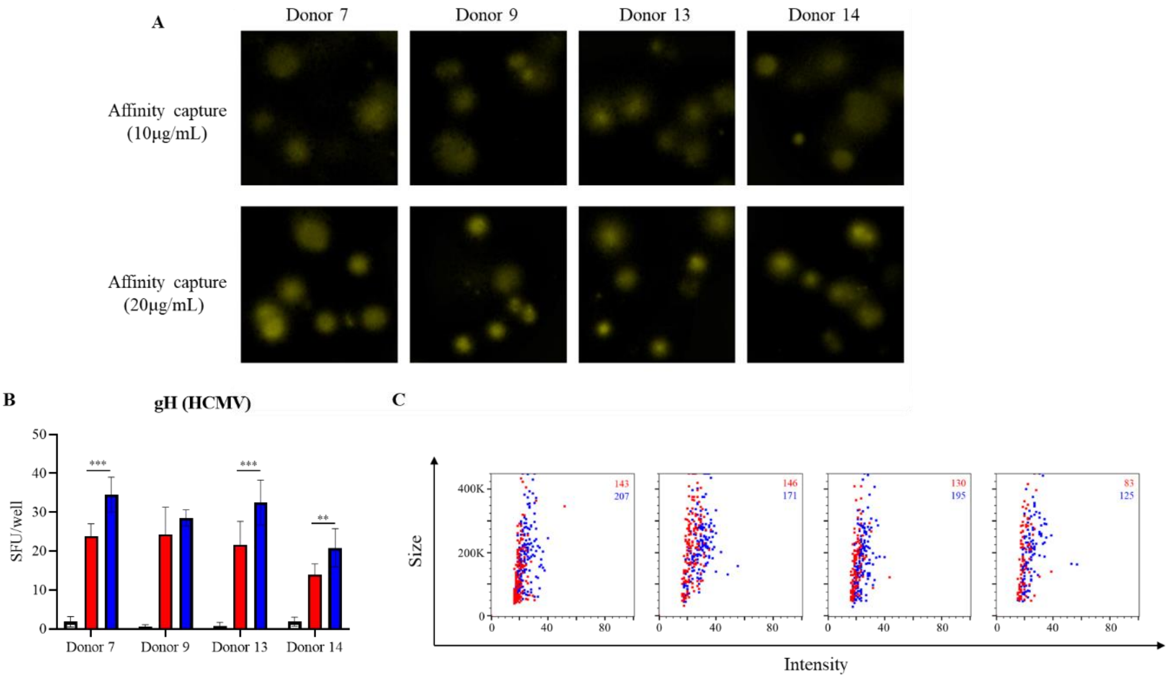

Supplementary Figure S1. Improved detection of gH-specific ASC through affinity capture of coating antigen. Human PBMC were in vitro stimulated and evaluated for antibody-secreting (ASC) reactivity against the gH pentamer complex protein from HCMV. (**A**) Representative wells images depicting antigen-specific IgG+ ASC in wells coated through affinity capture using the genetically encoded hexahistidine (6XHis) tag. Magnification and contrast enhancements were uniformly performed on all images to aid their visualization in publication. (**B**) Mean SFU/well (± SD) in assay wells in which the gH pentamer complex was affinity captured at 10 μg/mL (red) or 20 μg/mL (blue). SFU/well (± SD) in control wells in which hexahistidine (6XHis) peptide (2 μg/mL) was affinity captured (white). Significant differences in SFU/well were determined using an ANOVA with Sidak's post-hoc test (** $p < 0.01$, *** $p < 0.001$). (**C**) gH-specific FluoroSpots were merged into FCS files and visualized as bivariate plots. FluoroSpots originating from assay wells in which the gH pentamer complex was coated at 10μg/mL (red) or 20μg/mL (blue) are shown as overlays. The combined number of FluoroSpots detected in replicate wells for each of the respective donors is indicated in the inset.

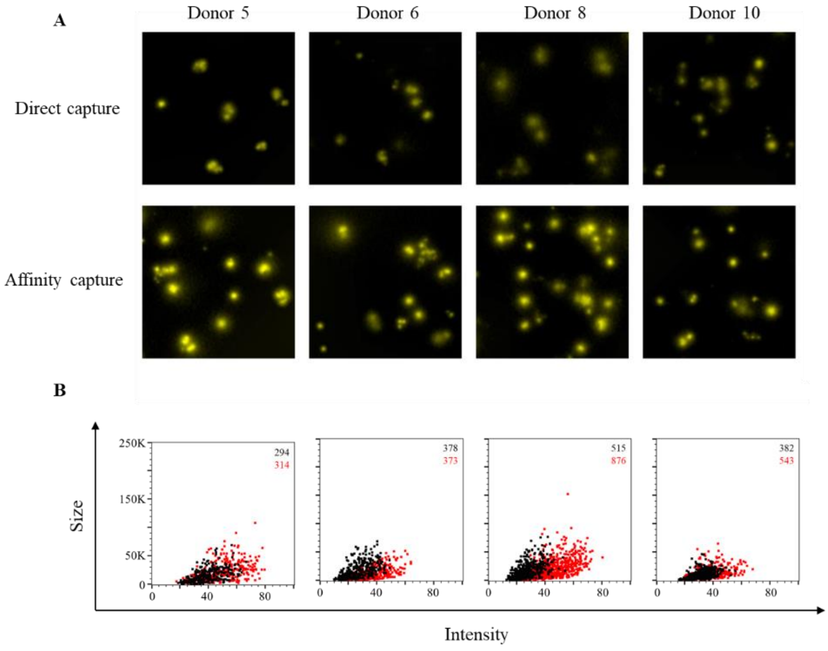

Supplementary Figure S2. Improved sensitivity of TX/12 rHA-specific ASC through affinity capture of coating antigen. PBMC were in vitro stimulated and evaluated for antibody-secreting (ASC) reactivity against recombinant hemagglutinin protein representing the A/Texas/50/2012 (TX/12) H3N2 vaccine strain. (**A**) Representative wells images depicting antigen-specific IgG^+ ASC in wells coated directly with 10 μg/mL of TX/12 rHA or through affinity capture using the genetically encoded hexahistidine (6XHis) tag. Magnification and contrast enhancements were uniformly performed on all images to aid their visualization in publication. (**B**) TX/12 rHA-specific FluoroSpots were merged into FCS files and visualized as bivariate plots. FluoroSpots originating from assay wells in which TX/12 rHA was directly captured on the membrane (black dots) or through affinity capture (red) are shown as overlays. The combined number of FluoroSpots detected in replicate wells for each of the respective donors is indicated in the inset using the same red/black color code.
